# Is it really a duplication cyst? Hypothesizing with insufficient data

**DOI:** 10.1590/1516-3180.2018.030006082019

**Published:** 2019-11-07

**Authors:** Dimitrios Sfoungaris

**Affiliations:** I MD, PhD. Assistant Professor, First Department of Pediatric Surgery, Aristotelion University of Thessaloniki, Thessaloniki, Greece.

Dear Editor,

The majority of digestive tract duplications that cause symptoms do so during early infancy.^1^^,^^2^ Cases with adult onset are very rare and present with a combination of signs and symptoms that include abdominal pain, intestinal obstruction and gastrointestinal bleeding.^3^ One such case was described by Huang et al.,^3^ and they followed on with a survey of the literature that revealed 20 additional adult cases, published up to July 2017. Furthermore, the feature of their case that is even more interesting is that the lesion developed on the antimesenteric border of the ileum, thus constituting an absolute rarity among cases of intestinal duplication, since these lesions have been reported to develop on the mesenteric border of the intestine.^1^^,^^2^^,^^4^

However, from carefully reviewing the authors’ findings, we have serious doubts about whether the lesion described is indeed a digestive tract duplication. The size of the lesion was not mentioned, but we can assume from the pictures that it is about 15 cm long in its dilated and edematous condition. Its location was reported to be 150 cm distally to the Treitz ligament, but its distance from the ileocecal valve was not mentioned. Assuming that the length of the jejunoileum in adults while alive is 258 cm on average,^4^ it would be possible for the lesion to arise around 100 cm proximally to the ileocecal valve. Interestingly, both the size and site of the lesion conform well with the characteristics of Meckel’s diverticulum, which may be as long as 56 cm,^5^ and which in 28% of the cases is located between 91 and 167 cm proximally to the ileocecal valve.^4^

From the pictures, we perceive that the lesion did not share a common wall with the native intestine, and that it emerged from its antimesenteric border as a true diverticulum, surrounded by a seromuscular layer. The authors did not mention anything about its communication with the native intestine’s lumen, or about its blood supply, even though both of these are important features for characterization of the lesion.^4^ Both cystic and tubular duplications shared a common wall with the native intestine and, regarding the type of tube, a communication would usually exist at one or both ends of the duplication.^1^^,^^2^ As an exception to this rule, a type of duplication cyst called the neurenteric cyst also exists: this is separate from the intestine but is attached in some way to the vertebrae and/or is accompanied by vertebral anomalies.^1^^,^^2^ However, no such anomalies were mentioned.

In conclusion, the imaging and operative data that are presented in this report do not support the diagnosis of an intestinal duplication. Instead, they fit very well with the much more common Meckel’s diverticulum, which in this case was infected, a complication more commonly occurring in adults,^5^ like the patient described in the report.
